# Transcatheter Mitral Valve Replacement With LAMPOON

**DOI:** 10.1016/j.jaccas.2025.105284

**Published:** 2025-10-01

**Authors:** Julien Feghaly, Naji Maaliki, Yixin Zhang, Mohamed Abdul Qader, Daniel Soffer, Ali Zgheib, Thomas Zeyl, Erol Belli, Valentin Suma, Calvin Choi

**Affiliations:** a Division of Cardiology, University of Florida College of Medicine–Jacksonville, Jacksonville, Florida, USA; b Division of Cardiothoracic Surgery, University of Florida College of Medicine–Jacksonville, Jacksonville, Florida, USA

**Keywords:** mitral valve, valve replacement, computed tomography, imaging

## Abstract

**Background:**

Left ventricular outflow tract (LVOT) obstruction is a potentially fatal complication of transcatheter mitral valve replacement (TMVR), particularly in patients with anatomically high-risk features. LAMPOON (laceration of the anterior mitral leaflet to prevent outflow obstruction) is a transcatheter electrosurgical technique developed to mitigate this risk by modifying anterior mitral leaflet anatomy prior to valve deployment.

**Objective:**

We describe our institutional experience with LAMPOON as a preventive strategy in TMVR cases with a high predicted risk for neo-LVOT obstruction.

**Methods:**

This case series includes patients with high-risk anatomical features who underwent TMVR with LAMPOON. Variations of the LAMPOON technique, including antegrade and tip-to-base approaches using the “flying-V” electrosurgical wire configuration, were employed based on individual anatomy.

**Conclusions:**

All patients underwent successful anterior mitral leaflet laceration, with no significant LVOT obstruction. Antegrade and tip-to-base LAMPOON techniques are safe and effective adjuncts to TMVR in patients at high risk of LVOT obstruction.

## Graphical Abstract

**Figure:**
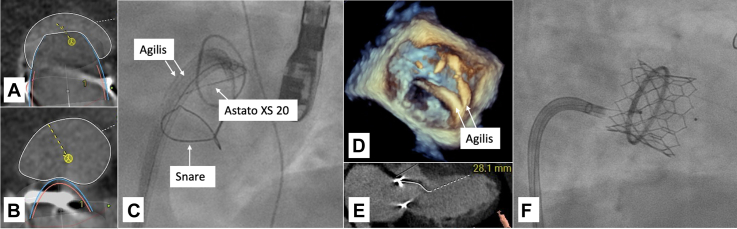

Mitral valve disease is a leading cause of cardiovascular morbidity and mortality globally. Transcatheter mitral valve replacement (TMVR) has emerged as a treatment option for patients with mitral stenosis or regurgitation who are considered at high surgical risk. However, left ventricular outflow tract (LVOT) obstruction remains one of the most feared and fatal complications of TMVR, especially in anatomies with a long anterior mitral leaflet (AML), narrow aortomitral angles, left ventricular basal septal hypertrophy, or bulky mitral annular calcification (MAC).^1^^,^^2^

**Take-Home Messages**

• LAMPOON is a transcatheter electrosurgical technique developed to mitigate neo-LVOT obstruction risk by modifying anterior mitral leaflet anatomy prior to TMVR.
• Antegrade and tip-to-base LAMPOON techniques are safe and effective adjuncts to TMVR in patients at high risk of LVOT obstruction.

LVOT obstruction occurs because of displacement of the AML toward the interventricular septum during valve deployment, narrowing the neo-LVOT and compromising blood flow. The incidence varies depending on anatomy and TMVR type: it is highest in the valve-in-MAC procedure (10%-40%) and lower in valve-in-ring and valve-in-valve procedures.^3^ Even with contemporary planning tools, up to 89% of potential TMVR candidates are excluded owing to high risk of LVOT obstruction.^1^

LAMPOON (laceration of the anterior mitral leaflet to prevent outflow obstruction) is a catheter-based technique that mimics surgical leaflet resection to preserve outflow tract patency. The technique has evolved from a complex retrograde approach to simplified antegrade and tip-to-base iterations, using electrosurgical laceration and guidewire modifications such as the “flying-V” configuration.^1^^,^^4^^,^^5^ This report presents our institutional experience with LAMPOON in TMVR, highlighting the antegrade and tip-to-base techniques and their role in expanding safe TMVR access to high-risk patients.

## Case 1: Valve-in-Ring TMVR

A 73-year-old woman with a history of mitral valve annuloplasty with 28-mm Physio ring, atrial fibrillation, and coronary artery bypass, presented with worsening dyspnea, chest pressure, leg edema, and fatigue in the setting of recurrent heart failure admissions.

Transesophageal echocardiogram (TEE) had demonstrated normal ejection fraction (50%-60%) and severe mitral regurgitation due to chordal rupture. She was deemed at high risk for surgical reoperation and was better suited for TMVR. Consideration was given to perform alcohol septal ablation to increase the neo-LVOT area. However, given her recurrent heart admissions, the plan was to proceed directly with TMVR.

**Figure 1:**
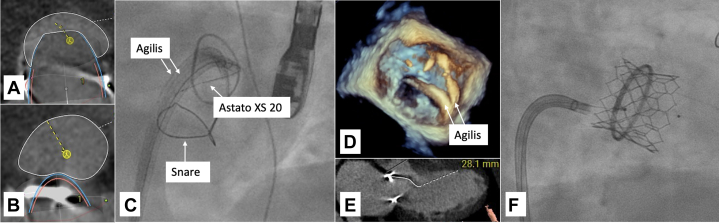
Imaging for Case 1 (A) CTA of neo-LVOT 207 mm^2^ without LAMPOON. (B) CTA rendering of neo-LVOT 388 mm^2^ with LAMPOON. (C) Fluoroscopy of antegrade LAMPOON. (D) Three-dimensional TEE of antegrade LAMPOON. (E) Anterior mitral leaflet length 28.1 mm. (F) Fluoroscopy showing valve-in-ring TMVR with 26-mm Edwards S3 at the time of subsequent paravalvular leak closure. CTA = computed tomography angiography; LAMPOON = laceration of the anterior mitral leaflet to prevent outflow obstruction; LVOT = left ventricular outflow tract; TEE = transesophageal echocardiogram.

Computed tomography angiogram determined a ring orifice area of 362 to 401 mm^2^. Therefore, a 26-mm Edwards S3 was chosen. With rendering using the 26-mm valve, we determined that the neo-LVOT area would be 207 mm^2^; additionally, the AML was 28 mm long. Given the length of the AML and a potential neo-LVOT area increase to 388 mm^2^ with AML electrosurgical laceration, we opted for TMVR with LAMPOON (Figures 1A, 1B, and 1E).

Trans-septal puncture was performed using a Baylis VersaCross RF system, followed by septostomy performed with a 14-mm balloon. Antegrade LAMPOON was performed with positioning of 2 Agilis steerable catheters (Abbott) across the septum. Under TEE guidance, we performed A2 scallop transversal with a JR4 guide in the atrial side of A2 and an EBU 3.0 (Medtronic) along with a 25-mm gooseneck snare on the ventricular side. The AML was crossed with an Astato XS 20 (Asahi Intecc), followed by laceration with electrocautery at 70 W pure cut, along with pulling and steady infusion of 5% dextrose fluid in both guides. Given the patient's anatomy and lack of prohibitive annular calcification, antegrade LAMPOON allowed for precise A2 scallop laceration as well as avoiding arterial access and aortic instrumentation and minimizing embolic risk. After successful LAMPOON, we proceeded with valve-in-ring TMVR using the 26-mm Edwards S3 (Figures 1C, 1D, and 1F). Further TMVR balloon dilation was limited owing to mitral ring shape and dimensions, and a posterolateral paravalvular leak was noted.

Follow-up transthoracic echocardiogram (TTE) demonstrated a well-seated mitral valve prosthesis, with laminar flow in the LVOT with no obstruction. The patient was discharged on postoperative day 3 and returned subsequently for paravalvular leak closure.

## Case 2: Valve-in-Valve TMVR

A 60-year-old woman with a history of bioprosthetic mitral valve replacement, tricuspid valve repair, coronary artery disease, atrial fibrillation, hypertension, and chronic kidney disease presented with worsening dyspnea, hypoxia, and edema.

TTE demonstrated a normal ejection fraction (55%-60%) and a well-seated bioprosthetic mitral valve with restricted leaflet opening and a transmitral gradient of 11 mm Hg at a heart rate of 86 beats/min, suggestive of significant prosthetic mitral valve stenosis. Additionally, the patient had a pulmonary artery systolic pressure of 65 mm Hg, further limiting her surgical candidacy. TEE confirmed thickening and calcification of the prosthetic leaflets, with a mean gradient of 12 mm Hg at a heart rate of 89 beats/min, and a mitral valve area of 0.9 to 1.0 cm^2^ by planimetry.

**Figure 2:**
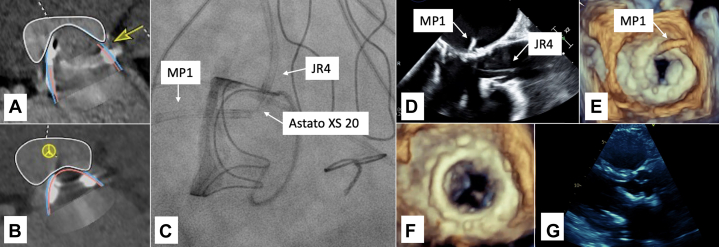
Imaging for Case 2 (A) CTA of neo-LVOT 227 mm^2^ without LAMPOON. The prior bioprosthetic mitral valve (yellow arrow). (B) CTA rendering of neo-LVOT 337 mm^2^ with LAMPOON. (C) Fluoroscopy of tip-to-base LAMPOON. (D) Two-dimensional TEE of tip-to-base LAMPOON. (E) Three-dimensional TEE of tip-to-base LAMPOON. (F) Three-dimensional TEE after tip-to-base LAMPOON. (G) TTE parasternal long-axis view showing LVOT after TMVR with 29-mm Edwards S3, with LVOT peak gradient 3 mm Hg. TMVR = transcatheter mitral valve replacement; TTE = transthoracic echocardiogram; other abbreviations as in Figure 1.

As the patient was deemed to not be a surgical candidate, the plan was to proceed with TMVR. Computed tomography angiogram determined that the bioprosthetic mitral valve had a diameter of 27 mm, and the patient would be best suited for a 29-mm Edwards S3. With rendering using the 29-mm valve, we determined that the neo-LVOT area would be 227 mm^2^ without modification of the bioprosthetic valve leaflets. Conversely, if we were to perform LAMPOON, the neo-LVOT area would increase to 337 mm^2^ (Figures 2A and 2B).

Trans-septal puncture was performed, followed by septostomy using a 14-mm balloon. We then proceeded with tip-to-base LAMPOON, where an Agilis steerable catheter, along with a balloon-tipped swan catheter, were used to cross the mitral valve, through which an R350 wire was advanced and then snared from the ascending aorta using a 25-mm gooseneck snare. The R350 was then externalized via the left femoral artery. Then, a 6-F JR4 guide was advanced via the left femoral artery, and a 6-F MP1 guide was advanced via the right femoral vein, both meeting below the bioprosthetic mitral valve leaflet.

The mid segment of an Astato XS 20 wire was scraped, exposing the core, then bent to 120°, forming the flying-V configuration. The R350 was exchanged for the Astato XS 20, and the laceration was performed with electrocautery at 70 W pure cut, along with pulling and steady infusion of 5% dextrose fluid in both guides. After the successful LAMPOON, we proceeded with valve-in-valve TMVR using the 29-mm Edwards S3, without any complications (Figures 2C to 2F).

Follow-up TTE demonstrated a well-seated mitral valve prosthesis with anterior struts protruding into LVOT, without obstruction or increase in gradient. The patient was discharged home and was doing well at the 6-month follow-up.

## Case 3: TAVR and Valve-in-Ring TMVR

A 68-year-old man with history of aortic valve endocarditis after undergoing surgical 27-mm bioprosthetic homograft, mitral valve repair with a 30-mm Genesee ring, coronary artery bypass, stroke, renal disease on dialysis, and atrial fibrillation, presented with worsening dyspnea in the setting of heart failure exacerbation.

TEE demonstrated an ejection fraction of 55% to 60%, severe mitral valve ring stenosis with mean gradient 11 mm Hg at a heart rate of 76 beats/min, severe aortic valve bioprosthesis regurgitation, moderate tricuspid and pulmonary regurgitation, and right ventricular systolic pressure of 66 mm Hg. Invasive valve hemodynamic assessment determined a mitral valve mean gradient of 15 mm Hg with a mitral valve area of 1.4 cm^2^, and an aortic valve mean gradient of 31 mm Hg with an aortic valve area of 1.1 cm^2^ in the setting of severe aortic valve bioprosthesis regurgitation. He was deemed at high risk for surgical reoperation, and the plan was to undergo transcatheter aortic and mitral valve replacement.

**Figure 3:**
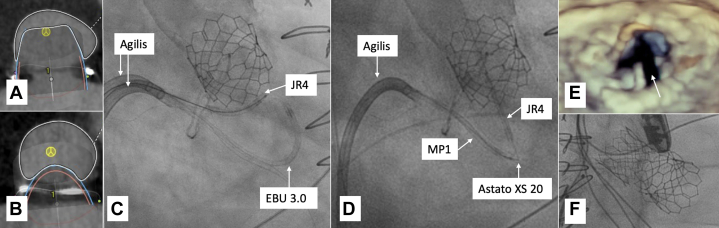
Imaging for Case 3 (A) CTA of neo-LVOT 249 mm^2^ without LAMPOON. (B) CTA rendering of neo-LVOT 426 mm^2^ with LAMPOON. (C) Fluoroscopy of antegrade LAMPOON. (D) Fluoroscopy of tip-to-base LAMPOON. (E) Three-dimensional TEE after LAMPOON showing anterior mitral leaflet laceration (yellow arrow). (F) Fluoroscopy showing valve-in-ring TMVR with 29-mm Edwards S3 and previously implanted TAVR with 26-mm Edwards Sapien S3. TAVR = transcatheter aortic valve replacement; TMVR = transcatheter mitral valve replacement; other abbreviations as in Figure 1.

The patient underwent successful valve-in-valve transcatheter aortic valve replacement (TAVR) with a 26-mm Edwards Sapien S3. His symptoms slightly improved after the TAVR, but he continued to have dyspnea and fatigue. Post-TAVR computed tomography angiogram determined a ring orifice area of 517 mm^2^. A 29-mm Edwards S3 was chosen for valve-in-ring TMVR. Computed tomography angiogram rendering determined that the neo-LVOT area would be 249 mm^2^; additionally, the AML was 26.5 mm long. Given a potential neo-LVOT area increase to 416 mm^2^ and to maximize safety, we opted for TMVR with LAMPOON (Figures 3A and 3B), as there was concern for potential anterior displacement of the mitral prosthesis toward the septum owing to interaction with the transcatheter aortic valve.

Trans-septal puncture was performed, followed by septostomy with a 14-mm balloon. The initial plan was for antegrade LAMPOON. Two Agilis steerable catheters were positioned in the left atrium. We attempted TEE-guided A2 scallop transversal using a JR4 guide in the atrial side of A2 and an EBU 3.0 on the ventricular side. We were not able to cross the AML, likely owing to anterior leaflet calcification. We then switched to tip-to-base LAMPOON. After the successful LAMPOON, we proceeded with valve-in-ring TMVR using the 29-mm Edwards S3. We were unable to initially advance the 29-mm valve across the septum and needed further septostomy with an 18-mm balloon, after which we successfully delivered the 29-mm Edwards S3 (Figures 3C to 3F). The atrial septal defect secondary to the septostomy was large, requiring closure with a 20-mm closure device.

Follow-up TTE demonstrated normal ejection fraction (65%-70%), well-seated aortic and mitral valve prostheses, and LVOT mild turbulent flow without any dynamic obstruction. The patient was discharged on postoperative day 3 with plans for outpatient follow-up.

## Discussion

LVOT obstruction is a devastating complication of TMVR and a leading cause of procedural ineligibility in patients with complex mitral anatomy. The LAMPOON procedure addresses this limitation by intentionally splitting the AML before valve implantation, thereby preserving LVOT patency.^1^^,^^2^ Our series supports the use of LAMPOON, particularly the simplified antegrade and tip-to-base variants, as a safe and effective adjunct in high-risk TMVR cases.

## Antegrade LAMPOON

Originally described using a retrograde aortic approach, LAMPOON required dual catheter navigation through the aortic and mitral valves—a process that is technically challenging and unsuitable for patients with mechanical aortic prostheses.^1^^,^^4^ Antegrade LAMPOON, performed through trans-septal access, resolves these limitations by using a femoral vein approach, enhancing catheter stability and eliminating the need for transaortic instrumentation.

**Figure 4:**
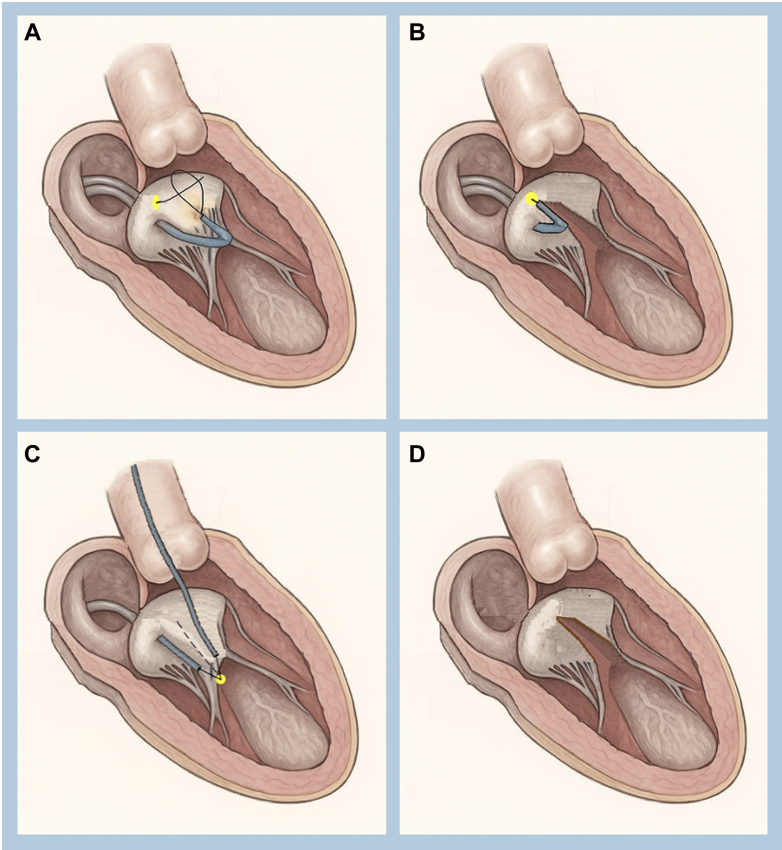
LAMPOON Techniques (A) Antegrade LAMPOON: Two Agilis steerable catheters are positioned in the left atrium. A gooseneck snare is positioned in the left ventricle. TEE-guided A2 scallop puncture using the tip of Astato XS 20 wire. (B) Antegrade LAMPOON: Anterior mitral leaflet laceration performed with electrocautery at 70 W pure cut. (C) Tip-to-base LAMPOON: Antegrade and retrograde guides in place, through which an Astato XS 20 wire is scraped, exposing the core and bent at 120°, forming the “flying-V” configuration. (D) After midline laceration of the anterior mitral leaflet in all LAMPOON techniques. Abbreviations as in Figure 1.

The antegrade technique has demonstrated technical success, with shorter procedural times and comparable hemodynamic outcomes relative to the retrograde approach.^4^ By aligning the traversal wire precisely with the A2 scallop under TEE and fluoroscopic guidance, the technique enables midline leaflet laceration, which is optimal for symmetric splaying and LVOT preservation^4^^,^^5^ (Figures 4A and 4B).

## Tip-to-Base LAMPOON and the Flying-V Configuration

In anatomies with a rigid surgical annular ring or bioprosthetic sewing ring, tip-to-base antegrade LAMPOON offers an even simpler alternative by eliminating the need for leaflet traversal. This variation is especially valuable in valve-in-valve and valve-in-ring TMVR, where the surrounding structure acts as a “backstop,” preventing injury to the aortomitral curtain or left atrial wall during laceration.^1^^,^^6^

The flying-V technique further refines leaflet laceration. By bending a short midsection of a 0.014-inch guidewire (eg, the Astato XS 20), electrosurgical energy is concentrated at the apex of the flying-V configuration, enabling a clean, controlled cut of the leaflet when traction is applied (Figure 4C).^1^^,^^4^ In our series, the flying-V technique enabled accurate midline leaflet laceration (Figure 4D), confirmed by intraprocedural imaging and postprocedural hemodynamic assessments.

These innovations address one of the key technical concerns of earlier LAMPOON iterations—eccentric or incomplete leaflet laceration, which could lead to residual LVOT narrowing or device interference.

## Preprocedural Planning and Safety Considerations

Accurate preprocedural planning is critical to LAMPOON success. Cardiac computed tomography is used to simulate virtual valve deployment and assess the neo-LVOT area. A projected neo-LVOT area of approximately 200 mm^2^ and AML length of >15 mm is considered higher risk and justifies the use of LAMPOON.^1^^,^^5^^,^^7^ LAMPOON has demonstrated favorable safety outcomes, with low rates of major complications such as stroke, perforation, or severe mitral regurgitation. In our series, as in prior reports, no patients experienced significant LVOT obstruction, and all patients underwent successful TMVR after leaflet laceration.^4^^,^^5^

Rescue (post hoc) LAMPOON has been described but is technically more challenging, typically requiring recrossing a deployed TMVR valve, which increases the risk of leaflet trauma and embolization. Alcohol septal ablation is an alternative to increase neo-LVOT area, but it requires delay for remodeling and carries risks of heart block and myocardial infarction.^4^ In urgent or borderline cases, pre-emptive LAMPOON remains the safer, more controlled strategy.

## Conclusions

Our case series demonstrates that antegrade and tip-to-base LAMPOON using the flying-V configuration is a reliable, efficient, and safe technique for preventing LVOT obstruction in high-risk TMVR patients. These advances significantly expand the population eligible for TMVR by overcoming the anatomical barrier of AML-related obstruction. As experience with this technique grows and planning tools improve, LAMPOON could become standard practice in anatomically complex TMVR cases.

## Funding Support and Author Disclosures

The authors have reported that they have no relationships relevant to the contents of this paper to disclose.
